# The Oral Microbiome and Head and Neck Cancer: A Narrative Review

**DOI:** 10.3390/cancers17172736

**Published:** 2025-08-23

**Authors:** Ewelina Golusińska-Kardach, Hariom Yadav, Shalini Jain, Michal M. Masternak, Wojciech Golusiński

**Affiliations:** 1 Department of Oral Surgery Periodontology and Oral Mucosa Diseases, Poznan University of Medical Sciences, 61-701 Poznan, Poland; 2 USF Center for Microbiome Research, Microbiomes Institute, College of Medicine, University of South Florida Morsani, Tampa, FL 33612, USA; hyadav@usf.edu (H.Y.); jains10@usf.edu (S.J.); 3 Center for Excellence in Aging and Brain Repair, Neurosurgery and Brain Repair, College of Medicine, University of South Florida Morsani, Tampa, 33612 FL, USA; 4 Burnett School of Biomedical Sciences, College of Medicine, University of Central Florida, Orlando, FL 32827, USA; michal.masternak@ucf.edu; 5 Department of Head and Neck Surgery, The Greater Poland Cancer Centre, Poznan University of Medical Sciences, 61-701 Poznan, Poland; wgolus@ump.edu.pl

**Keywords:** microbiome, head and neck cancers, oncology

## Abstract

In recent years, a growing body of evidence points to a link between alterations in the oral microbiome and the development and progression of head and neck cancer (HNC). At present, however, this highly complex relationship is only partially understood. In this context, there is a clear need to characterise and differentiate microbial profiles in patients with and without HNC to identify risk factors, prognostic biomarkers, and potential therapeutic targets. In the present narrative review, we synthesize the current evidence on the role of oral dysbiosis in HNC, with a particular focus on potential biomarkers and novel treatment and prevention strategies.

## Simple Summary

The oral microbiome plays a key role in the development of head and neck cancer (HNC). Studies showed that certain bacteria increase cancer risk, while others may be protective. However, the overall diversity of bacteria represents the key to health, since the balanced microbiome supports healthy mucosa, while dysbiosis—an imbalance of microbes—can promote cancer by triggering inflammation and weakening the immune response. Researchers are exploring the microbiome as a biomarker for early detection and as a target for new treatments, including probiotics and prebiotics. Though promising, more research is needed to fully understand these links and improve cancer care.

## 1. Introduction

The importance of the microbiome in the development and progression of numerous cancers, including head and neck cancer (HNC), has become increasingly evident in recent years [1,2]. The oral cavity harbours a diverse ecosystem of microorganisms (fungi, viruses, and bacteria), widely known as the oral microbiome. This humid, nutrient-rich environment, characterized by stable temperatures and pH levels, provides ideal conditions for microbial growth [3,4], as evidenced by the more than 700 bacterial species in this region, making it one of the most densely colonized areas in the human body [5,6,7].

While a balanced oral microbiota plays a crucial role in maintaining systemic and oral health, microbial imbalances—commonly referred to as dysbiosis—have been increasingly linked to various pathological conditions, including HNC [1]. Numerous studies have found an association between specific species of oral bacteria taxa and HNC [1,8,9,10], and oral dysbiosis has been proposed as an independent risk factor for its development [1,11].

**Figure 1:**
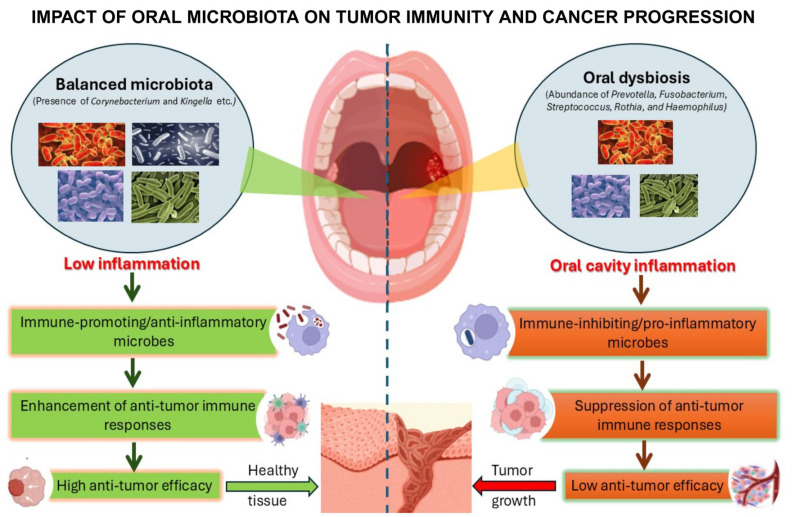
The Impact of Oral Microbial Homeostasis and Dysbiosis on Tumor Immune Surveillance.

Mechanistically, dysbiosis may trigger chronic inflammation and disrupt host cellular signalling pathways involved in cell proliferation, differentiation, and apoptosis [12]. Several oral commensal bacteria from the genera *Prevotella*, *Fusobacterium*, *Streptococcus*, *Rothia*, and *Haemophilus* have been associated with an increased risk of HNC [8,9,10,13,14,15]; by contrast, some bacteria—such as *Corynebacterium* and *Kingella*—appear to exert a protective effect [16] [Figure 1]. Research is currently underway to characterise and differentiate the microbial profiles in patients with and without HNC in an effort to identify prognostic biomarkers and potential therapeutic targets [2,17].

Although our understanding of the role played by the oral microbiome in oncogenesis is advancing rapidly, the relationship is complex and only partially understood [8]. In this context, the aim of the present narrative review is to synthesize current knowledge on the role of the oral microbiome in head and neck cancer, with a focus on emerging clinical applications, including potential biomarkers and novel treatment and prevention strategies.

## 2. Materials and Methods

### 2.1. Microbiome and the Risk of Head and Neck Cancer

This review is based on a comprehensive search of the PubMed, Scopus, and Web of Science databases. Search terms included combinations of: ‘oral microbiome’, ‘head and neck cancer’, ‘dysbiosis’, ‘immune microenvironment’, ‘HPV’, ‘probiotics’, and ‘microbial biomarkers’. We included peer-reviewed original research and reviews published in English between January 2005 and March 2025. Only studies involving human subjects or relevant animal models were considered. Editorials, letters to the editor, and case reports were excluded.

In healthy individuals, the oral microbiome constitutes a finely balanced ecosystem that plays a vital role in maintaining oral and systemic health. Disruption of this equilibrium, due to poor dietary habits, inadequate oral hygiene, alcohol and tobacco use, can lead to oral diseases of the mouth (periodontitis, dental caries, and gingivitis) and even oral cancer [5,18].

Emerging evidence has revealed significant associations between the composition of the oral microbiome and the risk of HNC [19]. Several studies have implicated an association between specific genera of oral bacteria—including commensal bacteria (*Streptococcus*, *Rothia*, *Fusobacterium*, *Haemophilus*, and *Prevotella*) and cancer onset [10], suggesting that oral dysbiosis is a contributing risk factor for HNC [8,9]. For example, one study, demonstrated that periodontitis, a condition indicative of oral dysbiosis, was associated with a 2- to 5-fold greater risk of developing oral cancer [6]. Another study found that tumour tissues from patients with HNC presented significant alterations in bacterial composition, including changes in the relative abundance of *Fusobacteria*, *Firmicutes*, *Actinobacteria*, and *Proteobacteria* [20]. Another investigation [21] identified significant differences between patients with oral cancer and healthy controls in the diversity of the oral microbiota and their metabolites. Interestingly, a case-control study [22] found that higher taxonomic alpha-diversity, the presence of oral fungi, and greater relative abundance of periodontal pathogens—particularly those belonging to the red and orange complexes—were associated with a lower risk of HNC.

Studies have consistently shown that the overall microbiome profile differs significantly between healthy individuals and those with HNC [1,23,24]. One recent study found a greater number of potential pathogens in the salivary microbiome of patients with oral cancer versus healthy individuals [25]. Other studies have identified increased quantities of *Lachnospiraceae* and *Eiknella* in the oral microbiota of HNC patients [8]. Conversely, certain bacteria (such as *Kingella and Corynebacterium*) have been associated with a lower risk of HNC [16].

Hamada et al. [10] categorized three major tumour-associated microbial signatures: *Fusobacterium*, *Prevotella*, and *Streptococcus*. Wang et al. performed a comprehensive analysis of microbiome signatures in HNC tissues [26], which revealed a correlation between the microbiome composition and certain clinicopathological characteristics (e.g., age, sex, tumour stage, and histologic grade). Similarly, Torralba et al. [27] reported an increased prevalence of *Fusobacteria*, *Bacteroidetes*, *and Firmicutes* in tumour tissues, suggesting that these microbiome signatures could serve as potential biomarkers to diagnose HNC and to monitor disease progression [28].

More recently, Kwak and colleagues analyzed saliva samples from three large, well-established cohorts to compare the microbiome profile of patients with and without HNC [1]. Those authors identified 22 bacterial species associated with the risk of developing head and neck squamous cell carcinoma (HNSCC), with some linked to an increased risk and others to a decreased risk. Notably, they identified 13 bacteria that were differentially associated with the risk of developing HNSCC, including *Leptotrichia*, *Streptococcus sanguinis*, *Prevotella salivae* and beta and gamma *Proteobacteria*. As those authors emphasized, it is important to analyze the microbial profile as a community rather than individually to increase the predictive value, which is why they developed a new tool—the microbial risk score (MRS)—to assess the risk of HNSCC. On this scale, a one standard deviation increase in the MRS is associated with a 50% higher risk of developing HNSCC.

Table 1 shows the bacterial species associated with an increased or reduced risk of HNC. The presence of these species, individually or as a group, could potentially serve as biomarkers to identify individuals at high risk. However, as Kwak et al. noted, analysing the microbial profile as a community (versus individually) provides much more information.

### 2.2. The Microbiome as a Modulator of Cancer: Systemic Inflammation and Immune Modulation

The microbiome plays an important role in modulating the development and progression of cancer through its influence on immune response and systemic inflammation, a highly complex relationship known as the cancer-microbiome-immune axis. Dysbiosis of the microbiome can promote an inflammatory microenvironment as pathogenic bacteria stimulate the production of pro-inflammatory cytokines and chemokines. For example, studies show that microbiota-driven IL-17 secretion modulates the immune tumour microenvironment (TME) across various cancer types [29]. The host microbiome contributes to immune homeostasis and aids in immune function by promoting colonization resistance as beneficial commensal bacteria outcompete pathogenic species in a healthy ecosystem [30].

Several studies have demonstrated strong associations between certain oral bacteria—particularly, *Fusobacterium nucleatum* (*F. nucelatum*) and *Porphyromonas gingivalis* (*P. gingivalis*)—and chronic inflammation [31]. These bacteria can interrupt the epithelial barrier and extracellular matrix, leading to an inflammatory microenvironment that contributes to both local and distant tumorigenesis. Moreover, these microorganisms modulate host immune response to promote cancer development through immunosuppression, T-cell responses, and cytokine dysregulation, thereby promoting tumour cell proliferation and migration [18]. Additionally, microbial metabolites associated with dysbiosis may suppress immune surveillance mechanisms contributing to immune evasion and tumour progression.

The oral microbiome may have both tumour-promoting and tumour-inhibiting characteristics, depending on the specific microorganisms and their interactions with the host and TME [32]. Some intratumoral microorganisms can enhance anti-tumour immunity by activating the STING signalling pathway, stimulating T cells and NK cells, and facilitating the creation of tertiary lymphoid structures within tumours [18]. The microbiome is essential for shaping and training key elements of the innate and adaptive immune systems; in turn, the immune system regulates and sustains vital aspects of the symbiotic relationship between the host and microbes [33].

In summary, the oral microbiome can significantly influence cancer development through complex interactions with inflammation and the immune system. However, our current understanding of these mechanisms remains limited. Further research is needed to develop new strategies for both prevention and treatment, which may include the targeted elimination of harmful bacteria in combination with the administration of beneficial bacteria [34].

### 2.3. Impact on the Tumour Microenvironment

The microbiome can influence the TME through various mechanisms, including alterations in immune cells, blood vessels, and peri-tumoral signalling molecules. In turn, these changes may promote recruitment of immune cells that support tumour growth or metastasis, potentially affecting treatment response [35]. Alterations in the oral microbiome can initiate and promote inflammation in the oral cavity to create a chronic inflammatory microenvironment that promotes carcinogenic processes, including proliferation, migration, apoptosis, cell growth, and differentiation into malignant phenotypes.

The oral microbiota can also alter the metabolic profile of the TME. For example, infection with *P. gingivalis* increases free fatty acid levels in the tongue and serum, thereby disrupting fatty acid metabolism [36]. Multiple oral bacteria species induce the production of pro-inflammatory cytokines (IL-1, IL-6, IL-8, TNFα) and other immune signalling molecules through the release of endotoxins and metabolic byproducts, which contributes to tissue degradation, inhibition of the antibacterial work of immune cells, and host tissue invasion [35]. In addition to direct modulation of immune signaling and inflammation, oral microbiota exert significant influence on the tumor microenvironment through the production of secondary metabolites. These include short-chain fatty acids (SCFAs) such as butyrate, propionate, and acetate, as well as reactive oxygen species (ROS), polyamines, and microbial toxins. SCFAs—especially butyrate—are known to have anti-inflammatory and anti-tumorigenic effects by acting as histone deacetylase (HDAC) inhibitors, thereby influencing epigenetic regulation of gene expression and promoting apoptosis in tumor cells [37,38]. Conversely, microbial metabolites such as polyamines (e.g., putrescine, spermidine) and hydrogen sulfide have been implicated in promoting cancer cell proliferation, angiogenesis, and immune evasion [39]. Lipopolysaccharides (LPS), secreted by Gram-negative bacteria, can further activate toll-like receptors (TLRs) and trigger pro-inflammatory signaling cascades within the TME, thereby facilitating a chronic inflammatory state conducive to tumor progression [40]. Thus, the metabolic activity of the microbiota plays a critical role in shaping the biochemical and immunological landscape of the TME, with context-dependent effects that may either suppress or promote oncogenesis.

There is a growing body of evidence indicating that the oral microbiome significantly affects the TME through multiple mechanisms, including inflammatory modulation, metabolic alterations, and immunomodulation playing a crucial role in the onset and progression of oral cancers.

### 2.4. Interaction Between the Microbiome and Viruses

The relationship between the microbiome and viruses in HNC is complex and not yet fully understood. However, current evidence suggests that this interplay may significantly contribute to the development and progression of cancer. The microbiota may promote virus-associated cancers by both direct and indirect mechanisms [41]. Alterations in the microbiome can directly enhance viral infectivity, while changes in gene expression may indirectly activate viral expression or induce inflammation, thereby synergizing with the tumorigenic effect of certain viruses. Bacteria and viruses may jointly contribute to the onset of oral cancer through chronic inflammation of the oral mucosa and TME, oxidative stress, modulation of host immune response (leading to immunosuppression and tumour progression), activation of inflammatory cell signalling pathways, and induction of epigenetic changes.

Several viruses, most notably human papillomavirus (HPV) and the Epstein-Barr virus (EBV), have been identified as key contributors in the development of oral cancer [42]. Oral dysbiosis can enhance susceptibility to HPV infection and/or modulate the immune response to HPV. Emerging evidence suggests that bacterial dysbiosis and HPV may both be heavily involved in malignant transformation [43]. Nevertheless, our current understanding of the interactions between HPV infection and the bacterial microbiota and their broader implications for human health remains limited [6,44].

While the interplay between the oral microbiome, viruses, and cancer is still being unravelled, ongoing research holds promise for uncovering the mechanisms through which viruses and the oral microbiota contribute to cancer onset, progression, and relapse. This knowledge may eventually lead to the development of new targets for prevention and treatment [45].

### 2.5. Relationship Between the Oral and Gut Microbiota

The microbiota of the oral cavity and the gastrointestinal tract are closely interconnected, forming a continuous microbial axis that significantly impacts human health [46]. Consequently, any discussion of the oral microbiome must necessarily consider the gut microbiome. While these microbial communities are distinct due to the presence of chemical barriers such as gastric and bile acids, under certain pathological and physiological conditions, these barriers can weaken, facilitating microbial translocation [47].

Advances in sequencing technologies have shed light on the complex relationship between gut dysbiosis and chronic inflammation, immune function, and the progression of cancer [46]. Translocation of oral pathogens to the gut (the oral-gut axis) has been observed in various disease states and is associated with gut dysbiosis and systemic inflammation [47], which creates a pro-inflammatory environment that can lead to immune dysfunction and tumorigenesis. Furthermore, metabolites derived from the gut microbiota (e.g., bile acids and short-chain fatty acids) are involved in modulating host immunity and inflammation thus influencing development and progression of HNC.

### 2.6. Treatment-Related Dysbiosis: Radiotherapy and Chemotherapy

Radiotherapy and chemotherapy can negatively impact the microbiota of both the gut and oral cavity, resulting in intestinal permeability and inflammation. In turn, these changes can reduce treatment effectiveness and increase adverse effects, such as oral mucositis and diarrhoea [48,49].

Studies have shown that the prevalence of opportunistic pathogens tends to increase after radiotherapy and chemotherapy [50]. Radiotherapy has been shown to disrupt gut microbial balance by increasing levels of harmful bacteria (e.g., *Enterobacteriaceae* and *Bacteroides*) while decreasing beneficial microbes such as *Bifidobacterium*, *Faecalibacterium prausnitzii*, and *Clostridium cluster XIVa* [48,51]. In the oral cavity, radiotherapy has been shown to decrease the abundance of some genera (*Haemophilus*, *Veillonella*, and *Granulicatella*), while increasing others such as *Lactobacillus* and *Enterococcus* [52].

Several studies have shown that supplementation with probiotics may help to restore the gut microbiota, thus improving immune response and, consequently, treatment outcomes [53]. One of the most commonly observed adverse effects in HNC patients undergoing radiotherapy is oral mucositis (OM). Several studies have evaluated the role of probiotics in preventing and treating OM. A recent study found that administering *Streptococcus salivarius* K12 can decrease the incidence of OM, as well as the duration and severity of mucositis [54]. A randomized clinical trial in patients with nasopharyngeal carcinoma found that probiotics were effective in preventing the development of OM in patients treated with chemoradiotherapy [55]. Certain probiotics, including *Bifidobacterium* and *Lactobacillus*, among others, may also be useful in the management of OM [37].

### 2.7. Biomarkers, Prevention, and Treatment

Numerous studies have found evidence that the microbiome can serve as both a predictive biomarker and a potential therapeutic target. Microbial modulation via the administration of prebiotics, probiotics, postbiotics, antibiotics, and dietary interventions offers potential for improving prevention, diagnosis, and treatment outcomes [56].

#### 2.7.1. Biomarkers

##### Predictive Biomarkers

The microbiome holds promise as a predictive biomarker for determining response to specific therapies, and for guiding the development of novel, personalized treatment strategies [57]. A wide range of bacteria have been associated with HNC and these specific microbial signatures may serve as biomarkers for HNC [23]. In this regard, an important contribution to the literature was made by Kwak and colleagues, who recently introduced the microbial risk score, a predictive framework integrating microbial profiles to estimate HNC risk [1]. The development of this tool exemplifies how far our understanding has advanced in the last few years, and, as datasets continue to expand, more refined tools will become available to further improve both diagnosis and prognosis.

#### 2.7.2. Immunotherapy

Immunotherapy has emerged as a robust anti-cancer treatment, although its efficacy is highly variable. A healthy microbiota can significantly improve the efficacy of chemotherapy and immunotherapy through immunomodulation and better drug metabolism [57,58].

Although multiple human studies have investigated the association between the composition of the microbiome and immunotherapy outcomes [59], specific research in terms of HNC remains limited. Nonetheless, interest in understanding the relationship between the composition of the microbiome and immunotherapy in HNC continues to grow, with studies highlighting the significant role of the microbiome in influencing cancer progression, treatment response, and toxicity [60].

A substantial body of evidence suggests that the gut microbiome is an important predictor of response to immune checkpoint inhibitors (ICI) in various cancers [61,62]. The gut microbiome profoundly influences the immune system and recent studies indicate that modulating the gut microbiome and microbial metabolites may improve response to ICBs [61]. Some of the proposed strategies to improve the efficacy of immunotherapy include faecal microbiota transplantation, probiotics, and dietary interventions [63].

Probiotics, preoperative immunonutrition, and microbiome modulation are being explored to enhance treatment efficacy and minimize side effects in HNC patients [24,64,65]. Clinical trials are being performed to assess the microbiome as a potential therapeutic target or biomarker in immunotherapy (NCT05375266).

The microbiome plays a crucial role in modulating immunotherapy outcomes in HNCs. Future research focusing on microbial signatures, metabolites, and targeted interventions could offer novel approaches to improve treatment efficacy and patient outcomes.

#### 2.7.3. Prevention

Probiotics have emerged as a promising approach in reducing the risk of oral cancer by modulating the oral microbiome, reducing systemic inflammation, and enhancing immune function [37,66,67].

Microbiome modulators such as prebiotics and prebiotics may help in cancer prevention by delivering healthy bacteria to the ecosystem, which then outcompete oncogenic pathogens such as *Helicobacter pylori* or *F. nucleatum* [68]. Probiotics can also metabolize certain dietary components, such as dietary fiber, to produce short-chain fatty acids (e.g., butyrate) with anticancer properties via histone deacetylase inhibition [18]. Studies show that these healthy bacteria help to regulate the oral and gut microbiome by producing important metabolites, most notably short-chain fatty acids; they also modify the pH in the gut, modulate the brain-skin-gut axis, and produce antimicrobial components [37].

Despite promising immunomodulatory and anti-tumorigenic effects, clinical data remain limited. More rigorous studies are needed to confirm the role of probiotics in the prevention of oral cancer.

#### 2.7.4. Anti-Cancer Treatments Targeting the Oral Microbiome

Data from several clinical trials suggest that targeting the oral microbiota could be a valuable complement to conventional oncological treatments, with the potential to improve treatment outcomes [69]. However, many challenges still remain. Probiotic formulations need to be standardized by accounting for inter-individual microbial variability and understanding strain-specific effects. Disruptions in the oral microbiome have been associated with a range of problems, including impairment of immune signalling pathways, weakening of epithelial barriers, alterations in cell cycles and apoptosis, genomic instability, metabolic changes, and angiogenesis [70]. Therefore, correcting dysbiosis through probiotics and prebiotics may help to restore homeostasis and inhibit cancer development, progression, and metastasis.

Recent research has revealed the importance of the oral and gut microbiome with regard to treatment outcomes. Strategies targeting these microbiomes seek to improve therapeutic efficacy and prognosis, while also reducing side effects. While promising, microbiome-targeted therapies face challenges such as variability in patient microbiota profiles and the lack of large-scale clinical validation. The focus of much current research is on identifying specific bacterial strains and/or metabolites that can serve as biomarkers or therapeutic agents for personalized cancer treatments.

Although research is ongoing and still largely preliminary, three main strategies have emerged, all of which are primarily aimed at modulating the microbiota: (1) administration of probiotics, prebiotics, and postbiotics, (2) performing fecal transplants from healthy donors [18,58], and (3) dietary interventions [18]. The current evidence base to support prebiotics and probiotics to enhance gut barrier function and modulate immune responses is limited but promising [71]. For example, clinical trials have demonstrated that certain probiotics, such as *Clostridium butyricum*, have been associated with better progression-free survival in cancer patients undergoing immunotherapy [2]. Additional studies are needed to validate and optimize these interventions.

### 2.8. Challenges and Future Directions

Research into the role of the oral microbiome in HNC is still in its infancy. Given the potential role of dysbiosis in the development and progression of HNC, further research is warranted to characterise the microbiome signatures associated with HNC to improve early diagnosis, prognosis, and to develop personalized treatment strategies to improve clinical outcomes [72]. In particular, future research efforts should focus on identifying the oral microbial profiles associated with cancer onset and progression and in identifying targeted therapies to alter the oral microbiota for clinical purposes.

A search of the ClinicalTrials.gov database reveals numerous clinical trials currently underway to assess the role of the microbiome in head and neck cancer. One such trial (NCT05837221) is being carried out to determine whether dysbiosis actively contributes to HNC and its underlying molecular mechanisms. In parallel, artificial intelligence is being applied to microbiome datasets to predict cancer risk, offering novel diagnostic and prognostic capabilities [73].

## 3. Conclusions

Oral dysbiosis plays a significant role in head and neck cancer by influencing inflammation and immune regulation. However, our understanding of these mechanisms remains limited, and more research is needed to develop novel prevention strategies and therapeutic interventions, which may include the targeted elimination of harmful bacteria combined with the administration of beneficial bacteria.

Large-scale microbiome studies that apply modern sequencing technologies are needed to define the microbial signatures associated with oral cancer. Strategies such as the administration of prebiotics, probiotics, and postbiotics may improve the effectiveness of conventional treatments but require validation in controlled clinical trials. Future innovations may include the administration of genetically-engineered oral bacteria for the prevention and treatment of oral cancer.

Moreover, integrating oral microbiome profiling into routine oncology care—for early detection, patient risk stratification, and therapy monitoring—holds substantial clinical promise. Clarifying regulatory pathways for probiotics and microbiota-targeted therapies will be essential to translate current findings into clinical practice. Ultimately, advancing our understanding of oral microbiota–host–tumor interactions could lead to more personalized and effective approaches to cancer prevention and treatment.

## Figures and Tables

**Table 1:** Bacterial species associated with a higher or lower risk of head and neck cancer.

Increased Risk of HNC (Pathological)	Reduced Risk of HNC (Beneficial)
*Streptococcus sanguiis*	*Corynebacterium*
*Rothia*	*Kingella*
*Fusobacterium nucleatum* *	*Fusobacterium nucleatum* *
*Haemophilus*	*Peptostreptococcus*
*Prevotella salivae*	*Clostridium butyricum*
*Leptotrichia*	
Beta and gamma *Proteobacteria*	
*Porphyromonas gingivalis*	
*Capnocytophaga*	
*Bacteroidetes*	
*Firmicutes phyla*	
*Lachnospiraceae*	
*Eiknella*	

* Fusobacterium may be both beneficial and pathological.
